# Towards More Nuanced Classification of NGOs and Their Services to Improve Integrated Planning across Disaster Phases

**DOI:** 10.3390/ijerph14111423

**Published:** 2017-11-21

**Authors:** Vivian L. Towe, Joie D. Acosta, Anita Chandra

**Affiliations:** RAND Corporation, 1200 S. Hayes Street, Arlington, VA 22202, USA; jacosta@rand.org (J.D.A.); chandra@rand.org (A.C.)

**Keywords:** disaster planning, nongovernmental organizations, disaster recovery, health security

## Abstract

Nongovernmental organizations (NGOs) are being integrated into U.S. strategies to expand the services that are available during health security threats like disasters. Identifying better ways to classify NGOs and their services could optimize disaster planning. We surveyed NGOs about the types of services they provided during different disaster phases. Survey responses were used to categorize NGO services as *core*—critical to fulfilling their organizational mission—or *adaptive*—services implemented during a disaster based on community need. We also classified NGOs as being core or adaptive types of organizations by calculating the percentage of each NGO’s services classified as core. Service types classified as core were mainly social services, while adaptive service types were those typically relied upon during disasters (e.g., warehousing, food services, etc.). In total, 120 NGOs were classified as core organizations, meaning they mainly provided the same services across disaster phases, while 100 NGOs were adaptive organizations, meaning their services changed. Adaptive NGOs were eight times more likely to report routinely participating in disaster planning as compared to core NGOs. One reason for this association may be that adaptive NGOs are more aware of the changing needs in their communities across disaster phases because of their involvement in disaster planning.

## 1. Introduction

The contribution of local nongovernmental organizations (NGOs) in the U.S. and abroad in disaster planning, response, and recovery has been well demonstrated [1]. For example, countries like China and Japan are frequently affected by natural disasters and therefore have developed similar strategies to engage NGOs across disaster phases [2,3,4,5]. NGO participation in relief activities and long-term support of victims is an advantage given that resources to support these types of activities can be stretched thin, particularly when disasters are increasing in frequency and growing in scale. Consequently, the U.S. government has integrated NGOs into national strategies, including the National Health Security Strategy and Implementation Plan 2015–2018 [6], which promotes a framework in which local, state, and federal agencies collaborate with NGOs and businesses to advance national health security. This framework strongly emphasizes integrated planning as an activity that includes NGOs, public health agencies, emergency management, faith-based groups and others across communities. Prior evidence of progress in better integration of planning includes an expansion of regional planning alliances and participation of organizations in coalitions for emergency planning. Also of note is NGO integration into the National Disaster Recovery Framework [7], which specifies the role of NGOs in pre-disaster planning, contributions from NGOs as part of Voluntary Organizations Active in Disaster (VOAD), and types of services they might provide during disaster.

NGOs provide support services in recovery, as was the case in Hurricane Katrina [8] and more recent disasters, such as Hurricanes Harvey, Irma, and Maria [9,10]. NGOs can also contribute to planning prior to disasters, particularly in building resilience to disasters [11]. Despite the gains NGOs have made in participating in disaster-related activities, many unanswered questions remain with regard to what constitutes optimal NGO integration into response and recovery efforts. Of particular importance are questions about how to match the needs of the event with services that specific NGOs offer, and whether and how certain NGOs can adaptively offer services that can change with the phase of disaster, given that the landscape of needs can evolve as conditions on the ground change (e.g., immediate response vs. long-term recovery). Answering these questions has become urgent given that, as specified in various national preparedness and security strategies, NGOs will continue to be consistently involved in disaster planning, response, and recovery, and the government is required to engage NGOs in such activities. However, NGO time and resources are generally limited. As such, we need more information to guide decisions about how to optimize NGO contributions [12] and to aid government agencies in their engagement of NGOs in local disaster efforts through better coordination, while more accurately funneling training and resources to community assets. 

Classifying NGOs, for example, as development organizations, public service contractors, grassroots organizations, etc., has been done in the past in order to distinguish roles and activities among NGOs [4]. While classifying NGOs seems like a reasonable first step toward optimizing their contributions to civil society, the overall process of classification is difficult due to the multifaceted nature of NGOs, for whom missions and activities often overlap classifications [13]. These classifications are even less useful for NGOs working in disaster-related activities because optimization of their contributions to disaster relies more on understanding what services NGOs provide, and not simply their organization type. While research has documented the variety of services and other contributions that NGOs have made during and after disasters [8,14,15], there is very little detail beyond this to operationalize a strategy that extracts the best value from NGO engagement in this field. Currently, NGOs are known to provide a broad range of services and with varying reliability and quality [16], but this could be improved if pre-disaster planning focused more on understanding how NGO roles and services should change over the disaster cycle from response to recovery and adapt with the changing needs of the community over the longer term.

To address the gap in our understanding of how NGO services can be optimized during the disaster cycle, we set out to gather key information through a survey of NGOs known to participate in disaster-related activities. The information was collected for two reasons: (1) to inform guidance for planners on what kind of data they should collect and maintain from NGOs about their services (i.e., in a database); and (2) to offer recommendations for NGOs about how they can best structure their service delivery in times of disaster, i.e., filling service gaps in the region. This is important because comprehensive information about NGO services is not readily available and requires planners to directly engage with all relevant NGOs, which can be time consuming.

## 2. Materials and Methods 

### 2.1. Study Design

The study team collected data from NGOs using a cross-sectional survey, which was in the field from March and April of 2015. The survey was conducted as part of a larger study to gain a better understanding of how NGOs participate in disasters across nations—in this case, the U.S. and China. However, only the U.S. data is analyzed and presented here. More information is provided about the larger study in a toolkit developed to facilitate better NGO–NGO and NGO–government coordination in disaster response and recovery [17].

The purpose of the survey was to answer three questions: (1) What services do NGOs provide during disaster response? (2) How do these disaster services differ from services provided during routine times or long-term disaster recovery? (3) How is the NGO provision of services during times of disaster associated with their regular participation in disaster planning in their communities? 

These research questions were developed to begin to test the model proposed by Acosta and Chandra (2013), which hypothesized that during a disaster, NGOs ramp up at least their routine services. We were interested in understanding the extent to which NGOs provide a set of *core services*—those that are critically important or central to fulfilling their organizational mission—during a disaster. If NGOs provide a set of core services, planners will understand what NGOs bring to the table, when their services are needed, and when NGOs will likely need to pull back support. As an example, an NGO whose mission it is to ensure that outreach and health workers are staffed where and when they are most needed in the community may report the core service as the identification and coordination of volunteers. However, if NGOs adopt new services during a disaster based on the needs of the community (we refer to these as *adaptive services*) by replacing or adding to their core services, this may strengthen their role in a disaster. But it would then be critical for them to engage disaster planners to ensure clarity on what services NGOs are offering and at what point during the response/recovery cycle these services will change. Gathering information from NGOs about their core and adaptive services can help emergency planners better engage NGOs by providing a new frame by which to understand and inventory the assets that NGOs bring to disaster response and recovery. 

### 2.2. Study Sample and Informed Consent

We assembled a list of NGOs for survey by reviewing member lists for the state chapters of Voluntary Organizations Active in Disaster (VOAD) and identifying contact information for VOAD member organizations. VOADs are typically coalitions of organizations covering a range of administrative geographies (e.g., city, county, regional, state, multi-state) whose mission is to mitigate and alleviate the impact of disasters. A link to an online survey was emailed to 576 potential VOAD organization respondents in March 2015 and three email reminders were sent to encourage participation. We asked respondents to complete the survey on behalf of their entire organization. Informed consent was obtained electronically prior to participation in the survey. Respondents were offered the option to receive a USD$20 gift card to reimburse them for the time spent completing the survey. The survey methods and content were reviewed and approved by the RAND Corporation’s Human Subjects Protection Committee. 

At the time the survey was closed in April of 2015, 241 organizations had responded to the survey, with a response rate of 42%, which is comparable to recent response rates for web survey administration [18]. Three surveys were missing responses to most or all questions and were dropped from the sample. An additional 18 respondents reported that they did not provide any services and were dropped from the sample, resulting in a final analytic sample of *N* = 220. Based on the respondents who answered a survey item on the organization’s geography, all U.S. regions were represented by the organizations: the Northwest (20.5%); Southwest (25.9%); Midwest (27.3%); Northeast (36.4%); and Southeast (41.8%). 

### 2.3. Measures and Statistical Analyses

All analyses were conducted using SAS 9.3 (SAS Institute, Cary, NC, USA).

#### 2.3.1. NGO Characteristics

We asked NGOs questions about whether their organization had members and how many they had, as well as about their membership structure (individuals vs. organizational members), history (e.g., length of existence), staffing situation (e.g., paid employees or volunteers), geography (e.g., rural vs. urban, U.S. region), and populations served (e.g., age, racial/ethnic groups, income level, etc.). Simple frequencies (means or percentages) were calculated for each of the NGO characteristics. 

#### 2.3.2. NGO Community Activities

We asked NGOs to select, from a pre-populated list, all of the activities their organization undertook to (1) build resilience in the community; (2) partner with government and nongovernment agencies; (3) facilitate transition from disaster response to recovery; and (4) engage and serve the community. We also asked NGOs to select, from a pre-populated list, (5) the types of information they used for planning and decision making during recovery. Simple frequencies were calculated for each of the activities or information types and are presented in Table 1; the ones with asterisks were found to be associated with the dependent variable “NGO routinely participating in disaster planning” at *p* < 0.05 in univariate logistic regression and are therefore used as covariates for the multivariable logistic regression described below.

#### 2.3.3. NGO Disaster Services

We asked NGOs to select, from a pre-populated list, all of the services they provided during: disaster response (within one month of the disaster), immediate recovery (one to three months after the disaster), and long-term recovery or routine times (more than three months after the disaster). Services they could select were: clothing; food services; animal services; warehousing (e.g., storing food, clothes, and other goods); mental health or counseling; spiritual support; job and unemployment assistance; housing (temporary or permanent); medical care; medication or pharmacy; case management, information or referral services; transportation; child services, child care, other child support; senior services; family violence (e.g., domestic violence, child abuse, interpersonal violence); immigrant services; financial assistance, including referrals for financial assistance; legal, insurance, and mediation services; construction or infrastructure development; volunteer opportunities; community liaison (e.g., representing community needs or interests); and preparing community members for the next disaster. This list was generated from a prior survey of NGO disaster services after Hurricane Sandy [19].

#### 2.3.4. Classifying NGO Disaster Services as Core or Adaptive

We then classified each disaster service as either *core* or *adaptive* by first calculating the proportion of NGOs that self-reported as offering the service during all three phases of disaster (disaster response, short-term recovery, and long-term recovery or routine times) and the proportion of NGOs that reported offering the service during only one or two phases of disaster. We then ran one-proportion *Z*-tests to determine if the proportions were different. If a higher proportion of NGOs reported that the service was offered during all three phases of disaster and the *Z*-test was significant at *p* < 0.05, the service was classified as “core”. If a higher proportion of NGOs reported that the service was offered during only one or two phases of disaster and the *Z*-test was significant at *p* < 0.05, the service was classified as “adaptive” (Table 2). Our reasoning was that if services shift by disaster phase and/or is not available at all times during disaster response and recovery, then it is not a core service for NGOs.

#### 2.3.5. Classifying NGOs as Core or Adaptive Organizations

**Figure 1:**
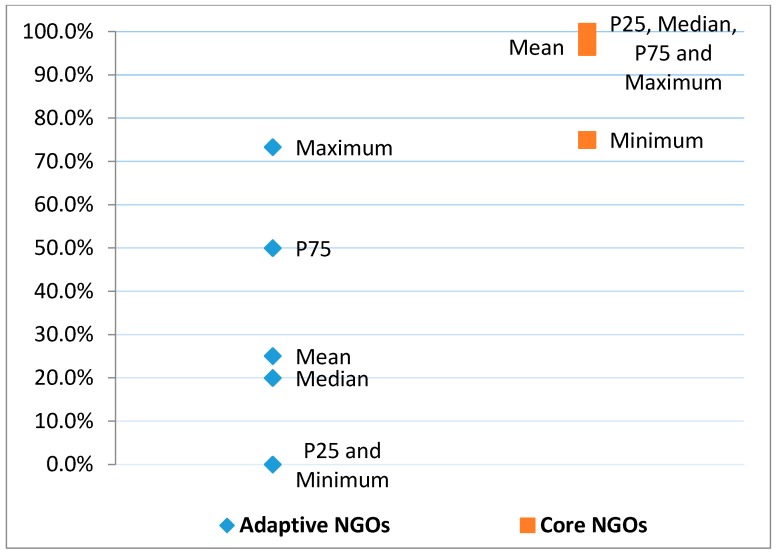
Sensitivity analysis for selection of the 75th percentile for core services as a cutoff for classifying core vs. adaptive NGOs.

In addition to the classification of each service as being core or adaptive, we also classified NGOs as being of core or adaptive types of organization overall by calculating the percentage of each NGO’s services that were classified as core. We conducted a sensitivity analysis on the core services variable to identify the optimal percentile cutoff for classification of NGOs as core vs. adaptive organizations. Optimal was defined as a cutoff in core services percentiles that resulted in minimally overlapping distributions of core service percentiles between core vs. adaptive organizations. Figure 1 shows that there is minimal overlap in core service percentiles between core and adaptive organizations when the cutoff is set at 75%. This lack of overlap in core service percentile means that the core NGO category is distinct in its definition compared to the adaptive NGO category and should therefore maximize our ability to detect differences between the NGO types. As a result, NGOs were classified as a core organization if 75–100% of their services were classified as core. NGOs with 74% or lower core services were classified as adaptive organizations. For one service type (financial assistance) that was classified as both core and adaptive, it was treated as core in this analysis.

#### 2.3.6. Identifying NGOs that Routinely Participate in Disaster Planning

Respondents indicated their level of agreement (strongly disagree to strongly agree) with the statement “Our organization routinely participates in disaster planning with government and nongovernmental partners in our community.” We then dichotomized this to create the dependent variable by categorizing respondents as being routinely involved in disaster planning (i.e., answered “agree” or “strongly agree” to the statement) or not being routinely involved in disaster planning (i.e., answered “disagree” or “strongly disagree” to the statement). 

#### 2.3.7. Modeling Predictors of an NGO Routinely Participating in Disaster Planning

A multivariable logistic regression examined the association between NGO type (core vs. adaptive) and NGO report of routine participation in disaster planning (the dependent variable) and included NGO participation in community activities (see Table 1) as covariates. Logistic regression was first used to identify statistically significant univariate associations (*p* < 0.05) between community activities and the dependent variable. All covariates significantly associated with the dependent variable were included in the multivariable logistic regression. Likelihood ratio (LR) testing was used to identify concise models in which each covariate was tested to see whether its inclusion resulted in a significantly different model compared to one without the covariate. Because the purpose of the model is to identify key activities associated with routine participation in disaster planning in communities, LR testing of models set significance at 0.10, which allows the inclusion of activities or drivers that are meaningful, but which might not be identified at strict significance levels. 

## 3. Results

### 3.1. NGO Characteristics

Nearly 84% of NGOs surveyed reported that they had been in existence for more than ten years. Over 60% indicated that they were an organization with members, either individual (e.g., congregants, grassroots volunteers), or organizational members (i.e., other organizations with formalized relationships to them). A vast majority of organizations reported serving children (81%), the elderly (86%), families (88%), racial and ethnic minorities (77%), low-income populations (85%) and non-English speaking populations (72%). In terms of past disaster experience, all NGOs surveyed reported some experience with disasters: 84% reported past participation in all three types of disaster phases: planning, response, and recovery, while 16% reported participating in only one or two types of disaster phases.

### 3.2. Whether NGO Services Are Core or Adaptive

Table 2 presents the percentage of NGOs that report service types offered during each of three disaster phases—disaster response, short-term recovery, and long-term recovery. Table 2 also presents the results of an analysis that classifies services as core (i.e., services that NGOs offer consistently across phases of disaster) or adaptive (i.e., services offered during only one or two phases of disaster). Service types classified as being core are comprised mainly of social services (e.g., family violence, senior services, immigrant services, etc.), while adaptive service types generally reflect services that are typically relied upon during disaster (e.g., warehousing, food services, clothing, etc.). Medical care was classified as an adaptive service, which is expected since most NGOs in this sample are not health clinics or other medical facilities.

### 3.3. Whether NGOs Are Core or Adaptive

We found that 120 NGOs were core organizations, meaning that they mainly provided the same services across all phases of disaster (see Table 2 for service types) and 100 NGOs were adaptive organizations, meaning they tended to provide different services across disaster phases. Core organizations provided an average of five types of services (SD 3.9) and 96% of those services were core services, whereas adaptive organizations provided an average of six types of services (SD 4.0) and only 27% of those services are core services (see Table 2 for which services were classified as core vs. adaptive). 

### 3.4. Whether Participation in Disaster Planning Differs for Core vs. Adaptive NGOs

Adjusted odds ratios for NGO type (the independent variable) and NGO participation in community activities (covariates) predicting NGO routine participation in disaster planning are presented in Table 3. Likelihood ratio testing results showed that the model in Table 3 contains only essential covariates; that is, the dropping of each covariate listed in Table 3 resulted in a significantly different model compared to one that included it. One key finding was in relationship to the primary question of NGO type and its relationship to the outcome of routine participation in disaster planning: adaptive NGOs were nearly eight times more likely to report routinely participating in disaster planning compared to core NGOs. Additionally, a specific set of key community activities was found to be independently associated with the outcome of routine participation in disaster planning: NGOs that reported training their program staff in emergency preparedness skills were 6.4 times more likely to report the outcome. NGOs communicating information to constituents/community members on where to go in an emergency were 6.8 times more likely to report the outcome.

## 4. Discussion

Findings from this survey indicate that NGOs provide many types of services to community residents and that these services can vary across the phases of disaster. Information from this survey was used to categorize NGO disaster services as either core or adaptive, as well as to categorize NGOs themselves as either core or adaptive based on the disaster services they delivered. Examples of core services include senior services, spiritual support, and providing volunteer opportunities. These services can be considered important and unique contributions upon which emergency planners can readily engage NGOs and depend on them to deliver throughout the disaster phase and possibly back to the steady state. These may also be services that, because of their durability, can be considered by policymakers as core for establishing contracts with NGOs, thus improving the region’s ability to quickly access those NGOs’ resources if a disaster occurs. Lack of resources has caused financial difficulties for NGOs because of long delays in the reimbursement processes (e.g., see our report on disaster case management after Katrina). If core services were under an earlier contract, a major hurdle to agile disaster response could be overcome. 

Our analyses also explored the concept of both adaptive services (e.g., food services, animal services, transportation, etc.) and adaptive organizations. The idea that NGOs can be flexible with their services and therefore responsive to the needs of their community is important for planners to consider, because those organizations may be the best ones to deploy for certain needs as conditions on the ground change. What is particularly compelling is that our analyses also showed that adaptive NGOs were significantly more likely to report routine participation in disaster planning in their communities than core NGOs. One reason for this association may be that adaptive NGOs are more aware of the changing needs in their communities between response, recovery, and routine times because of their involvement in disaster planning. However, there are a number of details about this finding that remain unclear. We are unclear about how NGOs participate in disaster planning and the extent to which the planning is integrated across key community sectors. We do not know the direction of the association between adaptive service models and disaster planning due to the cross-sectional nature of the survey. We also do not know about the quality or amount of adaptive services delivered by NGOs, which are just the type of services that are usually not offered across the disaster phases. However, based on the results of the survey, some NGOs are clearly aware that they change their services during disaster phases. Therefore, planners can leverage this and other findings from this paper to better engage NGOs. Future research should also delve more deeply into understanding how core and adaptive organizations may work differently with government agencies leading disaster response, and what the advantages and disadvantages are from both the NGO and lead agency perspective.

It is critical that planners effectively and efficiently engage NGOs—the findings from this paper suggest that planners should be asking NGOs about not only their services, but about how the services change across phases of disaster, what their capacity is for delivering adaptive services, what information they receive from the community that triggers the initiation of adaptive services, and their past experiences in delivering adaptive services. This paper offers a more robust and nuanced taxonomy upon which to classify NGOs and their services, which should support more reliable disaster resilience-building efforts. Gathering this information should allow planners to develop a more comprehensive landscape of disaster services and a more accurate timeline of when these services begin and end. Further, research should test the assumptions and findings from this analysis to observe how effective core and adaptive NGOs function in future disaster response and recovery.

This paper highlights one crucial area for improvement of NGO engagement during disasters: better classification of NGOs and their services. However, there are other areas as well, including improving trust and communication between NGOs and government agencies, for which work should be done between disasters. In the context of a trend of limited resources for addressing disasters, improving relationships and efficiency may be the best way toward well-coordinated cost-managed responses. 

### Limitations

The survey was cross-sectional in nature, so the logistic regression was not able to establish causality of NGO types or activities in relation to the outcome. Furthermore, the survey respondents were a convenience sample of NGOs known to participate in disaster-related work, so the results of the study are not necessarily generalizable to all types of NGOs. Results may be more representative of NGOs participating in disaster-related work, but we still recommend caution in generalizing to this NGO subgroup due to the non-random nature of the sample. In addition, the classifications that we developed for the NGOs (core and adaptive) were based mainly on the theory that service types and models were the most relevant drivers of how NGOs become involved in disaster-related work, but there are other ways to categorize NGOs that we did not explore. However, data on NGOs and their disaster experience are limited in their ability to specify the most relevant ways to categorize NGOs.

## 5. Conclusions

Overall, the diversity across NGOs identified here suggests that the government may need to provide different types and levels of support, training, and resources to ensure optimal NGO integration as partners with lead government agencies. For example, it may be useful for local planners to map all key NGO services to community geography and then work with groups of NGOs that provide specific services, using this framework of core and adaptive services. In this way, local planners will know exactly which NGOs will be working together on food services, and which will be working on temporary housing, etc. Given that NGOs will continue to play central roles in disaster response and recovery, their meaningful and appropriate integration into disaster planning is essential. The research presented here provides local emergency planners with more information about types of NGO disaster services and about key differences in NGO approaches to disaster service delivery. 

## Figures and Tables

**Table 1:** Frequencies of nongovernmental organization (NGO) participation in community activities and information used for planning and decision-making during recovery (*N* = 220).

NGO Community Activities/Information Types	NGOs (*N* = 220)	
*Resilience building activities*	N	%
Train our program staff in emergency preparedness skills *	171	77.7%
Serve on a committee or volunteer group dedicated to community preparedness or engagement (neighborhood council, city or county committee, local Community Emergency Response Team (CERT) team or disaster council) *	170	77.3%
Make sure constituents/community members have information about emergencies *	162	73.6%
Educate our constituents/community members that disaster preparedness is part of overall wellness planning *	140	63.6%
Create connections between community members for social support during difficult times	127	57.7%
Make sure constituents/community members have information on where to go in an emergency *	119	54.1%
Advocate for your constituents/community with government partners	109	49.5%
Refer community members to needed financial support services	104	47.3%
Refer community members to needed educational/training services	104	47.3%
Help fill gaps in unmet needs for individuals/families experiencing isolated emergency events (residential fires, for example)	103	46.8%
Identify gaps in services in your community for government partners to address *	94	42.7%
Assist partner NGOs in locating and obtaining funding for needed programs *	74	33.6%
Make sure constituents/community members have their health needs attended to on a routine basis (physical and/or psychological)	33	15.0%
Make sure constituents/community members have information on where to go in an emergency	119	54.1%
*Partnership activities*		
Have strong partnerships with other nongovernmental organizations	212	96.4%
Have good awareness of what our nongovernmental partners will bring to a disaster *	200	90.9%
Have strong partnerships with government agencies *	191	86.8%
*Activities to facilitate transition from disaster response to recovery*		
Identify partners for recovery activities in advance	146	66.4%
Have enough resources to provide in recovery	119	54.1%
Have plans that outline recovery protocols	109	49.5%
Train staff in recovery services (e.g., long term financial planning, long term mental health needs) *	87	39.5%
*Engaging and serving the community*		
Identify needs of affected residents	165	75.0%
Engage community leadership in disaster activities *	133	60.5%
Share important recovery information with residents in the community *	128	58.2%
Supported residents emotionally	124	56.4%
Help with broader community development (e.g., resilience, sustainability)	108	49.1%
Help rebuild damaged houses or infrastructure	99	45.0%
Inform the media on disaster recovery progress or activities *	88	40.0%
Supported residents financially	76	34.5%
Provide resources for mold cleanup	74	33.6%
Physically assist with mold cleanup	58	26.4%
Share community information with the disaster services contractors *	54	24.5%
Provide education on mold	48	21.8%
Expand/establish a local Community Emergency Response Team (CERT)	45	20.5%
Provide medical care to residents	21	9.5%
*Information used for planning and decision making during recovery*		
Training materials or tools on disaster recovery *	135	61.4%
Support or guidance we have received from other NGOs in the community	128	58.2%
Grant guidance describing recovery needs	51	23.2%

* These items had statistically significant univariate associations (*p* < 0.05) with the dependent variable “NGO routinely participating in disaster planning” discussed below.

**Table 2:** NGO core and adaptive service types by disaster phase (*N* = 220).

Service Type	Phase during Which Service Type Is Offered			Core/Adaptive Service Type *
	Disaster Response (% NGOs)	Immediate Recovery (% NGOs)	Long-term Recovery/Routine Times (% NGOs)	
Family violence (e.g., domestic violence, child abuse, interpersonal violence)	2.7%	3.2%	2.7%	Core
Senior services	10.5%	8.6%	6.8%	Core
Immigrant services	8.2%	8.2%	5.9%	Core
Volunteer opportunities	58.6%	52.7%	53.2%	Core
Community liaison (e.g., representing community needs or interests)	40.5%	37.7%	34.1%	Core
Job and unemployment assistance	3.6%	3.6%	4.5%	Core
Spiritual support	37.7%	30.5%	27.3%	Core
Legal, insurance and mediation services	4.1%	4.1%	3.6%	Core
Case management, information or referral services	33.6%	35.9%	34.5%	Core
Financial assistance	27.7%	29.1%	25.5%	Core/Adaptive
Child services-child care, other child support	8.6%	6.4%	5.5%	Adaptive
Mental health or counseling	21.4%	19.1%	18.2%	Adaptive
Preparing community members for the next disaster	32.3%	32.7%	42.3%	Adaptive
Warehousing (e.g., storing food, clothes, and other goods)	19.1%	15.0%	10.5%	Adaptive
Medication or pharmacy	7.3%	4.5%	2.7%	Adaptive
Construction or infrastructure development	18.6%	20.0%	25.5%	Adaptive
Food services	34.1%	20.0%	15.5%	Adaptive
Animal services	8.2%	4.1%	2.7%	Adaptive
Transportation	13.2%	7.7%	5.5%	Adaptive
Clothing	22.7%	13.2%	9.1%	Adaptive
Housing (temporary or permanent)	16.8%	14.1%	13.2%	Adaptive
Medical care	7.7%	4.1%	2.7%	Adaptive

* If more NGOs reported that the service was offered during all three phases of disaster and the *Z*-test was significant at *p* <0.05, the service was classified as “core”. If more NGOs reported that the service was offered during only one or two phases of disaster and the *Z*-test was significant at *p* <0.05, the service was classified as “adaptive”.

**Table 3:** Adjusted odds ratios (ORs) for NGO Type and NGO community activities associated with NGO routine participation in disaster planning.

Covariates	β (SE)	OR	(95% CI)	*p*-Value
*Type of NGO*				
Is an adaptive NGO *	2.1 (0.6)	7.9	(2.3–26.3)	0.0007
*Resilience building activities*				
Train our program staff in emergency preparedness skills	1.86 (0.6)	6.4	(2.0–20.8)	0.002
Make sure constituents/community members have information about emergencies	1.0 (0.6)	2.8	(0.9–9.0)	0.08
Make sure constituents/community members have information on where to go in an emergency	1.9 (0.8)	6.8	(1.3–34.8)	0.02
Identify gaps in services in your community for government partners to address	1.4 (0.7)	3.9	(0.9–16.4)	0.06
*Activities to facilitate transition from disaster response to recovery*				
Train staff in recovery services (e.g., long term financial planning, long term mental health needs)	−1.2 (0.7)	0.3	(0.07–1.2)	0.09
*Information used for planning and decision making during disaster recovery*				
Training materials or tools on disaster recovery	2.0 (0.6)	7.6	(2.2–26.6)	0.001
*Engaging and serving the community*				
Share community information with the disaster services contractors	1.8 (1.1)	5.8	(0.6–52.1)	0.12

* Compared to being a core NGO.
